# Change not State: Perceptual coupling in multistable displays reflects transient bias induced by perceptual change

**DOI:** 10.3758/s13423-021-01960-7

**Published:** 2021-08-02

**Authors:** Alexander Pastukhov, Claus-Christian Carbon

**Affiliations:** 1 Department of General Psychology and Methodology, University of Bamberg, Bamberg, Bavaria Germany; 2 Research Group EPÆG (Ergonomics, Psychological Æsthetics, Gestalt), Bamberg, Bavaria Germany

**Keywords:** Visual perception, Multistability, Perceptual coupling, Switch transient, Selective attention

## Abstract

We investigated how changes in dynamic spatial context influence visual perception. Specifically, we reexamined the perceptual coupling phenomenon when two multistable displays viewed simultaneously tend to be in the same dominant state and switch in accord. Current models assume this interaction reflecting mutual bias produced by a dominant perceptual *state.* In contrast, we demonstrate that influence of spatial context is strongest when perception *changes*. First, we replicated earlier work using bistable kinetic-depth effect displays, then extended it by employing asynchronous presentation to show that perceptual coupling cannot be accounted for by the static context provided by perceptually dominant *states*. Next, we demonstrated that perceptual coupling reflects transient bias induced by *perceptual change*, both in ambiguous and disambiguated displays. We used a hierarchical Bayesian model to characterize its timing, demonstrating that the transient bias is induced 50–70 ms after the exogenous trigger event and decays within ~200–300 ms. Both endogenous and exogenous switches led to quantitatively and qualitatively similar perceptual consequences, activating similar perceptual reevaluation mechanisms within a spatial surround. We explain how they can be understood within a transient selective visual attention framework or using local lateral connections within sensory representations. We suggest that observed perceptual effects reflect general mechanisms of perceptual inference for dynamic visual scene perception.

**Supplementary Information:**

The online version contains supplementary material available at 10.3758/s13423-021-01960-7.

Perception is the prerequisite of all cognitive acts and it must deliver a singular, determinate percept that represents the outside world despite intrinsically noisy and ambiguous sensory inputs. To solve this underdetermined problem, it strongly relies on additional sources of information, such as spatial context (Todorović, 2010). The latter has been extensively studied, and it has a profound effect on the perception of size (Murray et al., 2006), color (Hansen et al., 2007), or tilt (Gibson & Radner, 1937), to name just a few dimensions. The spatial context also strongly affects the perception of multistable stimuli, such as binocular rivalry or kinetic-depth effect (KDE; see Movie S1). These displays are perceptually unstable and give rise to two (or more) distinct interpretations so that observers’ perception endlessly alternates between them for as long as they view the stimulus. For these displays, an unambiguous static context alters switching dynamics by changing the balance of the perceptual competition, prolonging the dominance of the favored perceptual interpretation and curtailing that for the other one (Fang & He, 2004; Intaite et al., 2013; Klink et al., 2012; Sereno & Sereno, 1999).

The multistable displays are also an excellent tool for studying dynamic contexts. When several such displays are viewed simultaneously, their perception tends to be coupled together, so they are likely to be in the same dominant perceptual state and switch to a new state in accord (Attneave, 1968; Eby et al., 1989; Ramachandran & Anstis, 1983, 1985). This *perceptual coupling* reveals the mutual influence of individual multistable objects, with each one serving both as perceptual context and as a perceptual probe. The strength of perceptual coupling depends on the similarity of the bistable property (e.g., the orientation of the axis of rotation for KDE), objects’ proximity (smaller distances lead to stronger coupling), and ambiguity (disambiguation of one of the objects diminishes coupling (Grossmann & Dobbins, 2003; Pastukhov et al., 2018; but see Freeman & Driver, 2006; Klink et al., 2009). It is hypothesized to work via either a top-down mechanism (Grossmann & Dobbins, 2003) or local lateral connections (Klink et al., 2009). Its primary purpose is assumed to provide positive feedback to stabilize perception under challenging conditions such as multistablity.

Importantly, the perceptual coupling is thought to reflect a bias produced by a dominant perceptual *state* that facilitates the dominance of that same state in a neighboring object (Grossmann & Dobbins, 2003; Klink et al., 2009). However, as noted above, the perceptual coupling is reduced when one of the objects is disambiguated (Grossmann & Dobbins, 2003; Pastukhov et al., 2018). This creates a contradiction: If perceptual coupling reflects the influence of dominant perceptual states, the exogenous bias should stabilize the exogenously biased state, prolonging its dominance and enhancing rather than curtailing the perceptual coupling. Similarly, such state-based stabilizing feedback should slow down perceptual alternations, yet the perceptual coupling leads to faster switches (Pastukhov et al., 2018). Taking this into account, we hypothesized that perceptual coupling could be better explained by a transient mechanism that is activated by a *change* in perception rather than by its *state* and, therefore by the dynamics of the perceptual context. Below, we reexamine the phenomenon by replicating earlier work and determining that perceptual coupling cannot be explained by the influence of stable perceptually dominant states. Next, we demonstrate that coupling relies on transient interaction induced by perceptual switches that trigger a reevaluation of perception and produce transient bias favoring the new perceptual state in nearby spatial locations and objects. Finally, we show that this transient bias is a general consequence of perceptual changes as it is evoked by perceptual switches in both ambiguous and unambiguous displays.

## Methods

### Participants

Twenty-nine participants took part in Experiments 1 and 2, 27 females, two males; age range: 19–41 years. Only 15 people did experiments with coaxial and parallel layouts, whereas five did only the coaxial layout, and nine did only the parallel layout. Fifteen participants took part in Experiment 3, 12 females, three males; age range: 18–35 years. Two of the participants completed only two out of three experimental sessions, and three completed only one experimental session. These five participants were excluded from further analysis.

### Apparatus

Displays were presented on a Samsung SyncMaster 2233RZ monitor that had 47.5 × 29.5 cm visible area, with a resolution of 1,680 × 1,050 pixels and a refresh rate of 120 Hz. The viewing distance was 50 cm. Participants responded using a Cedrus RB-530 response box. Stimuli were generated using custom code and the PsychoPy library (Peirce et al., 2019).

### Displays

For a video example of stimuli for all experiments, please refer to Movie S1 in the Supplementary Materials.

**Fig. 1:**
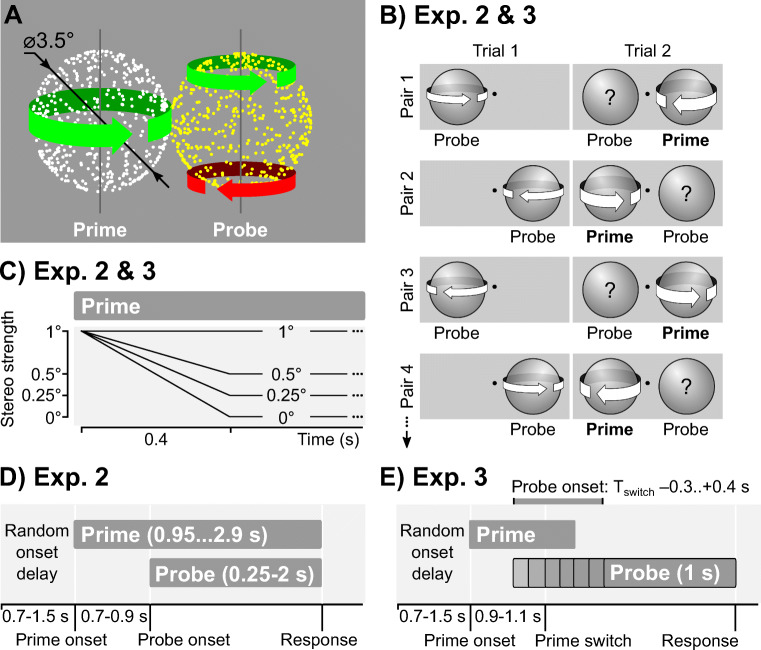
Stimuli and procedure. **a** Schematic display. The probe sphere (right) was fully ambiguous (arrows indicate that both directions of rotation were equally dominant), whereas the prime (left) was biased towards one direction of rotation (indicated by a single arrow). **b** Experiments 2 and 3. A block presentation sequence was split into pairs of trials (rows). In Trial 1, participants reported on the direction of the probe alone. In Trial 2, the prime appeared at the complementary position before the probe and was biased to rotate in the opposite direction to that reported for the probe on Trial 1. **c** Experiments 2 and 3. For the prime, the strength of the disambiguation cues was maximal at the onset and then linearly reduced to the predefined level over 400 ms. **d** Experiment 2. Presentation schedule for Trial 2 with both prime and probe spheres. The prime appeared before the probe and was biased to rotate in the direction opposite to that reported for the probe on the previous trial. Participants reported on the direction of rotation of both spheres. **e** Experiment 3. Presentation schedule for the Trial 2 for a s*witching prime*. A prime appeared before the probe and was biased to rotate in the same direction as reported for the probe on the previous trial. An inversion of on-screen motion in the prime sphere triggered a perceptual switch to the *opposite* direction of rotation. The probe sphere appeared −300–400 ms relative to the switch event. The prime disappeared 200 ms after the on-screen motion inversion. Participants reported on the direction of rotation of the probe sphere

Kinetic-depth effect (KDE) displays were an orthographic projection of a sphere (diameter 3.5°) that rotated around the vertical axis at the speed of 0.25 Hz. Each sphere consisted of 400 white or yellow semitransparent dots (diameter 0.08°) distributed randomly on the surface. The prime sphere in Experiment 3 was an exception, as it consisted of 50 dots to facilitate exogenously triggered reversals. In the *parallel* layout, the spheres were located to the left and the right of fixation (±1.75°, the spheres were touching) so that their axes of rotation were *parallel* (see Fig. 1a)*.* In the *coaxial* layout, they were presented above and below the fixation (±1.75°) so that their axes of rotation were *coaxial.* To facilitate perceptual grouping within a single object, one sphere was colored white, while the other one was yellow (Pastukhov et al., 2018). Spheres alternated their color on every block.

## Experiment 1. Continuous presentation

Experiment 1 consisted of 16 blocks generated from four levels of disambiguation, two locations of the prime (left and right for the *parallel* layout, above and below for the *coaxial* layout), and two directions of rotation of the disambiguated prime sphere (left and right).

The direction of rotation of the *prime* sphere was disambiguated via stereoscopic depth cues (anaglyph presentation). The projections for two eyes differed in their orientation around the vertical axis by 0° (fully ambiguous), 0.25°, 0.5°, or 1°. The strength of the disambiguation cues was constant throughout the entire presentation.

Each block started with a random onset delay (0.7–1.5 s) and lasted for 1 minute. Participants viewed the two spheres and continuously reported on their perceptual states. For the *parallel* layout, we used the following mapping: *left* or *right* button, if both spheres rotated, respectively, to the left or the right; *up* button, if left sphere rotated to the right and right sphere to the left (described as “into the screen”); *down* button, if left sphere rotated to the left and right sphere to the right (described as “out of the screen”); the round middle button, if the perception was unclear (approximately 0.2% of total block time). For the *coaxial* layout, participants were instructed to press *left* or *right* button if both spheres rotated, respectively, to the left or the right; *down* button whenever they perceived one of the counterrotation states; the round middle button, if the perception was unclear (approximately 0.1% of total block time).

### Experiment 2. Brief asynchronous presentation

Experiment 2 consisted of eight blocks. Each block consisted of 64 trials with four levels of disambiguation (0°, 0.25°, 0.5°, or 1°, same as in Experiment 1), four probe durations (0.25 s, 0.5 s, 1 s, or 2 s), and two locations of the prime (left and right off the fixation, same as in Experiment 1). The onset of each trial was randomly delayed by 0.7–1.5 s.

The block sequence was divided into 32 pairs of trials (see Fig. 1 b). During the Trial 1, only a fully ambiguous *probe* sphere was presented (presentation duration was 1 s, left column in Fig. 1b). Participants reported on its direction of rotation, informing us about the current dominant rotation direction at that location. Prior work showed that an ambiguous probe at this location tends to rotate in the same “default” direction (Knapen et al., 2009) unless its perceptual state is influenced by other factors, such as the dominant state of the prime in our case. During the subsequent Trial 2 (right column in Fig. 1b), the *prime* sphere appeared at the complementary position before the probe and was strongly biased to rotate in the opposite direction to that reported for the probe on the first trial (see Fig. 1d). The ambiguity of the prime sphere was linearly reduced to the target disambiguation strength throughout 400 ms (see Fig. 1c). Once the probe appeared (stimulus onset asynchrony was 700–900 ms), both were presented for another 0.25 s, 0.5 s, 1 s, or 2 s and disappeared simultaneously. Participants reported on the *final* direction of rotation of both spheres using the same mapping as in Experiment 1. In the case of probe-only trials, they were instructed to use only *left, right*, and *unclear* options. Participants reported *unclear* perception in approximately 0.4% (probe only) and 2.1% (both spheres) of trials for the coaxial layout and in 0.1% (probe only) and 1.7% (both spheres) for the parallel layout.

### Experiment 3. Brief asynchronous presentation with an on-screen-motion reversal event

Experiment 3 used only the parallel layout and consisted of three experimental sessions. Each session consisted of 10 blocks with three prime conditions and 10 probe onset times combinations randomly mixed within each block. Session 1 had 20 trials for all three prime conditions (see below) so that ten probe stimulus onset asynchronies (SOA) were presented twice (60 trials in total). Sessions 2 and 3 used only the two switching primes conditions (see below) and had 40 trials per condition (four repetitions of each probe SOA, 80 trials per block). Please note that two participants completed only two sessions, and three participants completed only one session. They were excluded from the analysis, but the incomplete data sets are available at the online repository.

The procedure was similar to Experiment 2 with the same sequence of probe-only and prime-plus-probe pairs of trials (see above and Fig. 1b), but with a different prime presentation schedule (see Fig. 1e). Specifically, we used three prime presentation conditions: (1) *switching ambiguous prime,* (2) *switching biased prime*, and (3) *stable ambiguous prime* (condition identical to Experiment 2)*.* The *biased prime* (Condition 2) remained strongly biased (1°) throughout the entire trial. *Ambiguous primes* (Conditions 1 and 3) were strongly biased (1°) at the onset, and their disambiguation cues were reduced to zero throughout 400 ms, as in Experiment 2 (see Fig. 1c). *Stable ambiguous primes* (Condition 3) were initially biased to rotate in the direction opposite to that reported for the probe on the previous trial (as in Experiment 2). *Switching primes* (Conditions 1 and 2) were biased to rotate in the same direction as one reported for the probe on the previous trial so that the opposite direction of rotation would become dominant following a perceptual switch. For *switching primes*, the 2D on-screen motion was reversed 900–1,100 ms after the onset leading to a perceptual switch in the direction of rotation (Pastukhov et al., 2012). Probe onset was timed relative to the on-screen motion reversal (a hypothetical one, in case of *stable ambiguous primes*) with SOAs −300, −200, −100, −50, 0, 50, 100, 200, 300, and 400 ms. Prime disappeared 200 ms after the probe onset. The probe duration was always 1,000 ms (i.e., it disappeared 800 ms after the prime). Participants responded on the final direction of rotation of the probe (left/right/unclear), there were no *unclear* responses.

### Statistical analysis

Statistical analysis was performed in R, Version 4.0.0 (R Core Team, 2019) using tidyverse packages (Wickham et al., 2019).

For Experiments 1 and 2, we quantified the importance of individual fixed effects using the following measures. For each fixed effect term (referred to as β in figures), we computed its estimated mean value and a 95% credible interval, a range that contains 95% of values from the sampled posterior distribution (CI, also called compatibility interval). Additionally, each term’s importance was assessed by fitting a full model and the model without a term (a drop-one approach) and comparing these models via widely applicable information criterion (WAIC). The difference between the two models:

$$ \Delta WAIC= WAIC\left( full\ model\right)- WAIC\left( model\ without\ term\right), $$

is reported in the Supplementary Information. Lower values of WAIC indicate better expected out-of-sample deviance. Accordingly, negative values of ΔWAIC indicate that a full model is preferred over the model with the term. Also, ΔWAIC was used to compute each model’s relative weight (*W*) reported in figures (Bürkner, 2018). The two weights add up to unity so that values above 0.5 indicate that the fixed factor’s inclusion improves expected models’ prediction accuracy. Finally, we compared models with and without a term via Bayes factor (BF) with values above 3.2, 10, and 100 indicating, respectively, substantial, strong, and decisive support for the model with the term (Kass & Raftery, 1995).

For Experiment 1, we modelled the proportion of dominance time for individual perceptual states using beta family with a logit link and default priors (Bürkner, 2018). Specifically, we modelled the proportion of dominance times as:

$$ {P}_{dominant}\sim Stereo+ Strength+\left(1| Participant\right), $$

where *P*_*dominant*_ is a proportion of time the perceptual state was dominant (values were scaled so that the range was from 0.0005 to 0.9995 to avoid extreme values of 0 and 1), *Stereo = Strength > 0°*, *Strength* is the strength of disambiguation cues (0°, 0.25°, 0.5°, 1°); *Participant* identity was used as a random factor.

For experiment 2, we modelled the number of trials when both spheres or each sphere rotated in the direction of the bias using the binomial family with a logit link and default priors (Bürkner, 2018):

$$ {N}_{bias}\mid N\sim Strength+ Duration+ SOA+ Strength: Duration+\left(1| Participant\right), $$

where *N*_*bias*_ is the number of trials when the sphere(s) rotated in the direction of the bias, *N* – the total number of trials, *Strength* – the strength of disambiguation cues (0°, 0.25°, 0.5°, 1°), *Duration* – duration of the probe (0.25 s, 0.5 s, 1 s, 2 s), *SOA* – probe onset time relative to the onset time of the prime (0.7–0.9 s), *Strength:Duration* – an interaction term, *Participant* – random factor.

For Experiment 3, we assumed that the prime’s influence on the probe depends on an overlap of two critical time windows: (1) an exponentially decaying strength of the switch-induced transient bias, following the on-screen-motion reversal event, (2) exponentially decaying sensitivity of the probe perception following its onset. The second time window corresponds to the process of perceptual inference until the probe’s perception is resolved in favor of a particular state (Pastukhov, 2016). The prime’s influence is proportional to an area of an overlap of the two curves (see Fig. 4a). Mathematically, we approximated this as:

$$ {\displaystyle \begin{array}{c} Prime\ Influence=\mathit{\exp}\left(-\frac{T^{later\ event}-{T}^{bias}}{\tau_{bias}}-\frac{T^{later\ event}-{T}^{probe}}{\tau_{probe}}\right),\\ {}{T}_{later\ event}=\max \left({T}^{bias},{T}^{probe}\ \right),\end{array}} $$

where *T*^*bias*^ and *T*^*probe*^ are, respectively, bias and probe onset time relative to the trigger event, *T*^*later event*^ is a time of the later event in the trial, and τ^*bias*^ and τ^*probe*^ are, respectively, bias and probe decay time scales. We used independent bias onset and decay time constants for the two prime conditions, ambiguous and biased. We programmed a hierarchical Bayesian GLM in Stan (Carpenter et al., 2017) as follows:

$$ {\displaystyle \begin{array}{c}\ {N}_i^{bias}\sim Binomial\left({N}_i,{p}_i\right)\\ {} logit\left({p}_i\right)={\alpha}_P+{\beta}_{P,C}\cdotp PrimeInfluence\left({T}_P^{probe},{T}_{P,C}^{bias},{\tau}_P^{probe},{\tau}_{P,C}^{bias}\right)\\ {}\begin{array}{c}{\alpha}_P\sim Normal\left({\mu}^{\alpha },{\sigma}^{\alpha}\right)\\ {}{\beta}_{P,C}\sim Normal\left({\mu}_C^{\beta },{\sigma}_C^{\beta}\right)\\ {}\begin{array}{c}{T}_{P,C}^{bias}\sim Normal\left({\mu}_C^{T_{bias}},{\sigma}_C^{T_{bias}}\right)\\ {}{\tau}_{P,C}^{bias}\sim Normal\left({\mu}_C^{\tau_{bias}},{\sigma}_C^{\tau_{bias}}\right)\\ {}\begin{array}{c}{\tau}_P^{probe}\sim Normal\left({\mu}^{\tau_{probe}},{\sigma}^{\tau_{probe}}\right)\\ {}{\mu}_C^{T_{bias}}\sim Cauchy\left(50,50\right)\\ {}\begin{array}{c}{\mu}_C^{\tau_{bias}},{\mu}^{\tau_{probe}}\sim HalfCauchy\left(100,50\right)\\ {}{\mu}^{\alpha },{\mu}_C^{\beta}\sim Cauchy\left(0,10\right)\\ {}\ {\sigma}^{\alpha },{\sigma}_C^{\beta },{\sigma}_C^{T_{bias}},{\sigma}_C^{\tau_{bias}},{\sigma}^{\tau_{probe}}\sim HalfCauchy\left(0,10\right),\end{array}\end{array}\end{array}\end{array}\end{array}} $$

where the subscript *i* indicates the data row, subscript *P* is a participant index, subscript *C* is a prime condition index (1–biased, 2–ambiguous), *N*^*bias*^ is the number of trials when a participant reported that the probe rotated in the direction of the bias, *N* is the total number of trials, and *T*^*probe*^ is the probe onset time relative to the on-screen motion reversal trigger event. Following McElreath (2016), we used weakly regularizing Cauchy and half Cauchy priors centered at zero for most parameters, except for ${\mu}_C^{T_{bias}}$ that was centered at 50 (ms) and ${\mu}_C^{\tau_{bias}},{\mu}^{\tau_{probe}}$ that were centered at 100 (ms) based on prior work (Pastukhov, 2016). The model was sampled using four chains with parameters adapt_delta = 0.98 and max_treedepth = 15.

## Results

### Experiment 1

In our first experiment, we sought to replicate and extend earlier work on perceptual coupling, particularly the reduced perceptual coupling when one of the spheres is disambiguated (Grossmann & Dobbins, 2003). To this end, we presented two rotating spheres that were placed either side-by-side (parallel axes of rotation; see Fig. 1a) or one above the other (coaxial layout, see Supplementary Materials for results). The rotation of one *(prime*) sphere was disambiguated via stereoscopic depth cues, whereas the other *(probe*) sphere was fully ambiguous. The participants viewed the spheres for one minute and continuously reported on the direction of rotation of *both* spheres.

**Fig. 2:**
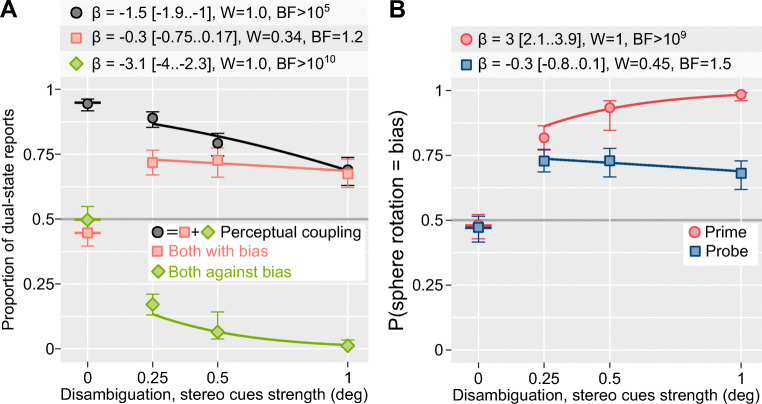
Experiment 1. **a** The proportion of time the two spheres rotated in the same direction overall ( *Perceptual coupling*), in the direction of the bias ( *Both with bias)* or against the bias ( *Both against bias*) as a function of stereo cues’ strength. **b** The proportion of time the individual spheres ( *Prime* or *Probe*) rotated in the direction of bias as a function of stereo cues’ strength. **a–b** Circles and error bars depict group mean and bootstrapped 95% bias-corrected accelerated confidence intervals. Solid lines show the prediction of a median model. The tables above the plot show the main effect of *stereo strength* on each dual state’s dominance (**a**) and direction rotation of a sphere (**b**); for further details, see Table S2 in the Supplementary Materials. β = mean estimate and 95% credible interval; W = relative weight of the full model compared to a model without the stereo strength term (two weights add up to 1, weight above 0.5 indicates the importance of the term); BF = Bayes factor for models with and without stereo strength term, value above 3.2, 10, and 100 indicating, respectively, substantial, strong, and decisive support for the model with the term. (Color figure online)

Our results were in complete agreement with the earlier work, as the spheres were strongly perceptually coupled—rotated mostly in the same direction—when both were fully ambiguous (see Fig. 2a; see Fig. S1 and Table S1 in the Supplementary Materials). Also, as in the original report, the proportion of time the participants reported corotation decreased significantly for strongly disambiguated primes. However, the analysis showed that this effect was explained solely by a decrease in the proportion of time the two spheres rotated together *against the bias*. Instead, the proportion of time the two rotated together *with the bias* remained constant despite increasing strength of disambiguation cues (there was an effect of the mere presence of the depth cues but not of their strength; see Table S1 in the Supplementary Materials). For the individual spheres, even though stereoscopic depth cues significantly increased the proportion of time the *prime* sphere rotated in the direction of the bias, there was no significant effect on the ambiguous *probe* (see Fig. 2b). In short, increasing the amount of time the biased state was dominant for the *prime* sphere did not increase the dominance of that same state in the *probe*. This observation challenges the idea that perceptual coupling comes from a perceptually dominant state (Grossmann & Dobbins, 2003).

### Experiment 2

**Fig. 3:**
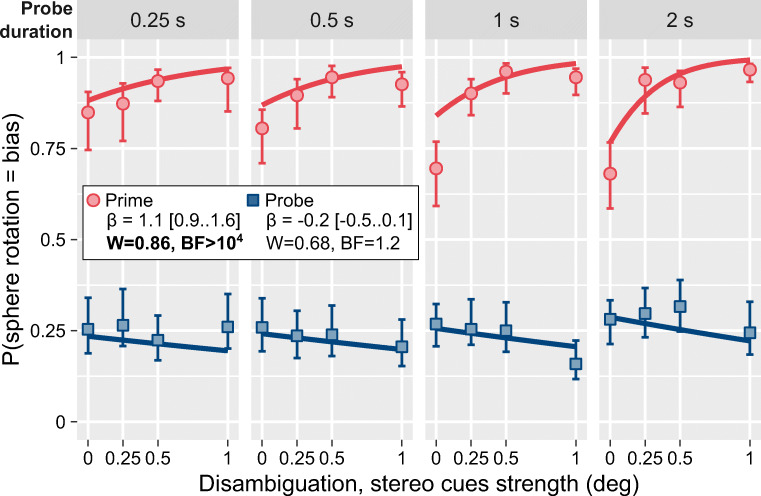
Experiment 2 (see also Fig. S2 in the Supplementary Materials). The proportion of trials when an individual sphere ( *Prime* or *Probe*) rotated in the direction of bias and opposite to the direction of rotation for the probe reported on the previous trial (see Fig. 2 for figure and legend details, and Table S2, in the Supplementary Materials, for full statistical analysis). (Color figure on line)

To confirm that state-based mechanisms cannot explain perceptual coupling, we performed our second experiment that employed a brief asynchronous presentation of *prime* and *probe* spheres. We hypothesized that for state-based mechanisms, if a prime sphere appears before the probe, the dominant perceptual state of the former should perceptually couple the latter biasing its onset perceptual state towards the same direction of rotation. For this, we split the presentation sequence into pairs of trials (see Fig. 1b). During the first trial, the participants viewed and reported only on the ambiguous *probe*, informing us about the current dominant direction of rotation at that location (i.e., the direction of rotation we would expect to be dominant again at this location on the subsequent trial; Knapen et al., 2009). During the subsequent trial, the prime sphere appeared at the complementary position before the probe (stimulus onset asynchrony was 700–900 ms) and was biased to rotate in the *opposite* direction to that reported for the probe on the first trial (see Fig. 1b–d). The probe’s delayed onset provided a perfect opportunity for bias from the prime’s dominant perceptual state to influence it. Thus, if perceptual coupling reflects an influence of a dominant state, we would expect prime to reliably bias the onset perception of the probe towards its dominant direction (the probability that the probe rotated in the direction of bias [*P(probe = bias) > 0.5*]. Conversely, a tendency of the probe to rotate in the same direction as on the previous trial and, therefore, opposite to the prime [*P(probe = bias) < 0.5*] would indicate a weak state-based perceptual coupling. We observed the latter, with uniformly low probabilities of the probe rotating in the direction of bias for all stimulus onset asynchronies and strength of the biasing cues (see Fig. 3). For the *Probe*, the probability did decrease slightly for stronger disambiguation cues, but this effect was not statistically significant. In short, the prime’s perceptual state had little influence on the perceptual state of the probe either during onset perception or continuous viewing (Experiment 1).

### Experiment 3

**Fig. 4:**
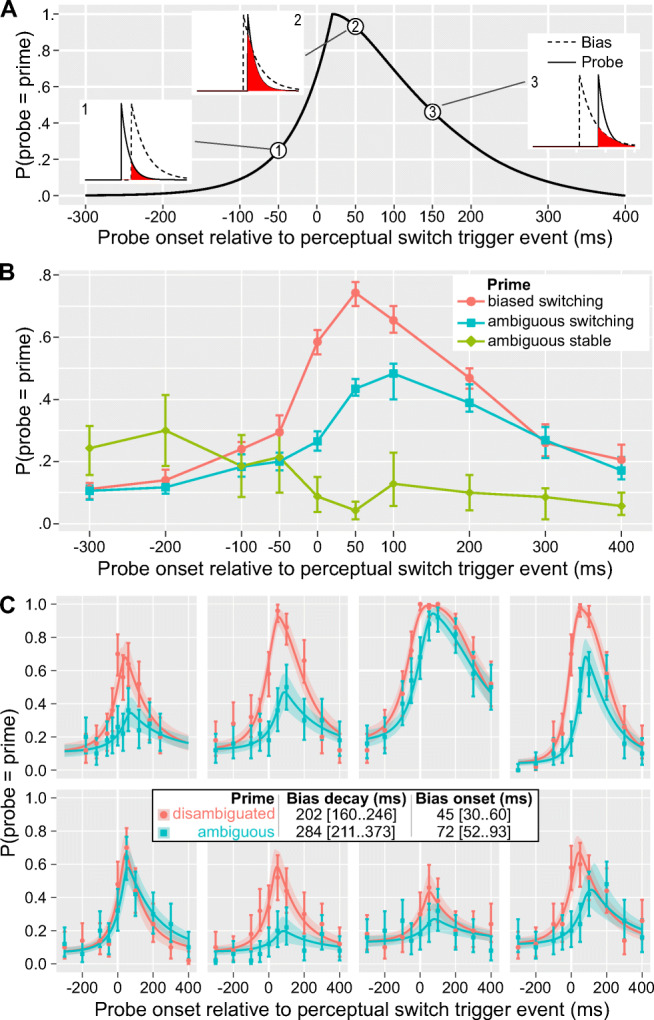
Experiment 3. **a** Schematic model. An illustration of the interaction between exponentially decaying sensitivity of probe perception after the onset (solid line in inset plots, example τ_probe_ = 50 ms) and exponentially decaying strength of the switched-induced bias (dashed line in inset plots, example τ_bias_ = 100 ms, bias onset 50 ms after the trigger event). The stronger influence of the prime on probe perception, reflected in higher *P*(probe = prime), corresponds to the larger overlap of the two time-windows (red area in inset plots). **b** Group average for the proportion of trials when the probe was reported to rotate in the same direction as the prime. Circles and error bars depict group mean and bootstrapped 95% bias-corrected accelerated confidence intervals, respectively. **c** Data and model fits for individual participants. The proportion of trials when the *probe* sphere rotated in the same direction as the prime, following the switch, and against the expected default rotation direction of the probe, as a function of the *probe* onset time relative to the on-screen-motion reversal event. Circles and error bars depict mean and bootstrapped 95% bias-corrected accelerated confidence intervals. Solid lines and stripes show the prediction of a median model and 95% credible interval. The legend inset show mean and 95% credible intervals for population-level bias decay and onset parameters. (Color figure online)

The alternative to the state-based bias is a transient mechanism that is active whenever an object *changes* its perceptual state. This transient influence does not need to be strong to influence perceptual dominance if it occurs at the right time (i.e., at the stimulus onset; Song & Yao, 2009) or when a currently dominant state is weakened by adaptation (Lankheet, 2006). To test this hypothesis, we replicated Experiment 2 using stable primes (as in Experiment 2) and extended it using *switching* primes. In the latter case, an on-screen motion reversal at a predefined time triggered a perceptual switch to the direction of rotation opposite to one reported for the probe on the previous trial. We hypothesized that if bias is transient and induced by a perpetual switch, we should see its effect only when the probe appears shortly before or after a perceptual switch (see Fig. 4a). Moreover, the attenuation of the prime’s influence on the probe should be proportional to decay times of both the transient bias and probe perception onset sensitivity.

The *stable ambiguous prime* condition replicated Experiment 2 and produced very similar results (see Fig. 4b, green line and diamonds). For the *switching primes*, both ambiguous (Fig. 4b, blue line and squares) and disambiguated (Fig. 4b, red line and circles), we found that perceptual switches biased the probe’s perceptual state most strongly if it was presented shortly after the on-screen-motion reversal.

To quantify this effect, we fitted data using a hierarchical Bayesian model, assuming that the strength of the prime’s influence reflects a degree of temporal overlap of the two exponentially decaying processes: 1) the transient change-induced bias of the *prime* and 2) the onset sensitivity of the *probe* perception (see Fig. 4a and Methods for details). The fits for individual participants are shown in Fig. 4c. The model indicated that the transient bias occurs shortly after the on-screen motion-reversal event, with the perceptual switch itself occurring no earlier than 20 ms after that (Pastukhov & Klanke, 2016), and decays rapidly (decay time constants 200–300 ms), see legend inset in Fig. 4c; see Fig. S3, and Table S3 in the Supplementay Materials. The bias was present for both ambiguous and unambiguous (disambiguated) primes but was weaker, occurred later, and decayed slower for the former. Thus, our results show that perceptual coupling works via a switch-induced mechanism that broadcasts a transient bias favoring the new perceptual state at nearby locations and objects.

## Discussion

We reexamined the perceptual coupling phenomenon when several multistable displays tend to be in the same dominant perceptual state and switch synchronously. In Experiments 1 and 2, we ruled out a hypothesis that mutual bias reflects the influence of the perceptually dominant state as was proposed in the prior research (Grossmann & Dobbins, 2003; Klink et al., 2009). Next, we demonstrated that the perception of bistable rotating spheres is influenced by a bias produced whenever one of them switches to a new dominant state. Moreover, we found that the transient bias was produced by perceptual switches in both ambiguous and disambiguated displays, showing that perceptual coupling is not specific to multistability and does not depend on stimulus ambiguity.

The mechanisms of change-detection play a crucial role in our daily lives (Simons & Rensink, 2005), triggering reevaluation of perception (Serences & Yantis, 2006), amplifying the change signal (Mehrpour et al., 2020), and ensuring, among other things, correct visual feature binding (Parto et al., 2021) and the possibility of rapid behavioral responses (Parto et al., 2021). Prior work indicated that these mechanisms are common to both unambiguous (Martinez-Trujillo et al., 2007) and multistable (Britz et al., 2009; Kanai et al., 2005; Kornmeier et al., 2009; Takahashi & Watanabe, 2010) displays. Our results offer further support for this link and demonstrate how multistable perception can characterize temporal and spatial properties of these mechanisms.

### Switch-induced perceptual coupling

Our results show that perceptual coupling is produced not by a dominant perceptual state, as was assumed in prior work (Grossmann & Dobbins, 2003; Klink et al., 2009), but by a *switch* to a new dominant perception. This idea is fully compatible with prior experimental evidence and allows for a parsimonious explanation of observations that are problematic for a dominant-state bias hypothesis. As noted in the introduction, the latter has no simple explanation of why perceptual coupling is reduced when one of the objects is disambiguated (Grossmann & Dobbins, 2003). Our results suggest that this is because rare perceptual switches in a disambiguated prime mean equally rare opportunities for it to influence probe via switch-induced transient bias. Conversely, the prime’s high perceptual stability makes it resistant to transient bias produced by frequent perceptual switches in the probe. Thus, although the strength of the transient bias itself is not affected by sphere disambiguation, the combination of these two factors leads to an apparent decrease of perceptual coupling during continuous viewing.

Another problematic finding is that perceptually coupled displays tend to switch more frequently than a single sphere (Pastukhov et al., 2018). The dominant-state bias hypothesis predicts longer dominance phases because the mutual stabilizing bias should increase the dominant state’s relative strength, biasing perception in its favor. In contrast, the switch-induced transient bias predicts observed shorter dominance durations for coupled displays because either object can initiate a joint perceptual switch, doubling the sources of perceptual instability.

In short, an idea of switch-induced transient bias offers a more parsimonious account for accumulated experimental evidence than a dominant state bias theory.

### Perceptual consequences of endogenous and exogenous perceptual switches

Perceptual switches produced the transient bias in both ambiguous and disambiguated spheres meaning that it is common to endogenous (spontaneous) and exogenous (physical) perceptual switches. The difference between the two is one of the critical questions in multistability research. On the one hand, prior research identified transient neural activity in certain frontal and parietal areas specific to spontaneous but not perceptually matched physical switches (Brascamp et al., 2018) but see (Kornmeier & Bach, 2012). On the other hand, the same meta-analysis revealed transient activity common to both types of switches in other areas associated with perceptual decision making and feedback mechanisms, such as the dorsolateral prefrontal cortex. Recent work by de Jong et al. (2020), using the intracranial recording, demonstrated that endogenous and exogenous switches rely on the same perceptual hierarchy, differing primarily in the order that these areas are activated.

Our results highlight another commonality between exogenously and endogenously induced switches by showing that both trigger the same mechanism of perceptual reevaluation. Taken together, this suggests that although spontaneous and physical switches may differ in how they are initiated, they tap into the same or shared processing networks and lead to qualitatively and, even, quantitatively similar perceptual consequences.

### Possible neural mechanisms of switch-induced transient bias

Prior research suggested top-down feedback as a possible mechanism for perceptual coupling (Eby et al., 1989; Grossmann & Dobbins, 2003). Selective attention, in particular, would be a prime candidate. It modulates both rates of perceptual switches (Pastukhov & Braun, 2007) and strength of perceptual coupling (Mareschal & Clifford, 2012), although it is not strictly necessary for either to occur (but see Dieter et al., 2016, on a special case of binocular rivalry). In this framework, a perceptual switch could activate a “circuit breaker” ventral frontoparietal network (Corbetta & Shulman, 2002), attracting attention and prompting a reevaluation of perception to reestablish coherent activity across regions of the visual system (Serences & Yantis, 2006). Here, a transient feedback signal would stabilize a new perceptual state (de Jong et al., 2020; Weilnhammer et al., 2013), affecting spatially adjusted populations with similar selectivity. The “spilling over” effect would reflect a larger receptive field size of neurons at the top of the processing cascade (Dumoulin & Wandell, 2008) and could be an integral part of perceptual inference over space (Spillmann et al., 2015).

Alternatively, a prior work modelled perceptual coupling via local lateral connections between similarly and oppositely tuned groups of neurons (Klink et al., 2009). Although this model assumed sustained bias, it is likely to be similarly capable with a switch-induced transient bias. Similarly, a model of binocular rivalry for ring displays, which is essentially a perceptual coupling display composed of multiple adjacent binocular rivalry stimuli, allowed for traveling waves of dominance using just a two-layer model with only local connectivity (Lee et al., 2005). These mechanisms could be like those that facilitate rapid contour integration in the primary visual cortex (Pack & Born, 2001; VanRullen et al., 2001). In the end, although we lean towards the selective attention hypothesis, it is up to future research to resolve this question.

## Conclusions

To conclude, we report that both endogenous and exogenous perceptual switches produce a transient signal that initiates perceptual reevaluation in the nearby locations. It is likely to reflect transient top-down feedback that stabilizes the new perceptual state of the changed object and biases nearby locations towards the new dominant state.

## Supplementary Information

ESM 1 (MP4 26868 kb)

ESM 2 (PDF 1157 kb)

## Declarations

All procedures were in accordance with the national ethical standards on human experimentation and with the Declaration of Helsinki of 1975, as revised in 2008, and were approved by the University of Bamberg via the local Ethic committee—Der Ethikrat der Otto-Friedrich-Universität Bamberg.
