# Supplementary material for: Change not State: Perceptual coupling in multistable displays reflects transient bias induced by perceptual change
Source: Psychon Bull Rev. 2021 Aug 2;29(1):97–107. doi: 10.3758/s13423-021-01960-7 (PMC8858312; doi:10.3758/s13423-021-01960-7)
Supplement: Supplementary file 2 — (PDF 1157 kb) [file 13423_2021_1960_MOESM2_ESM.pdf]

Electronic supplementary material

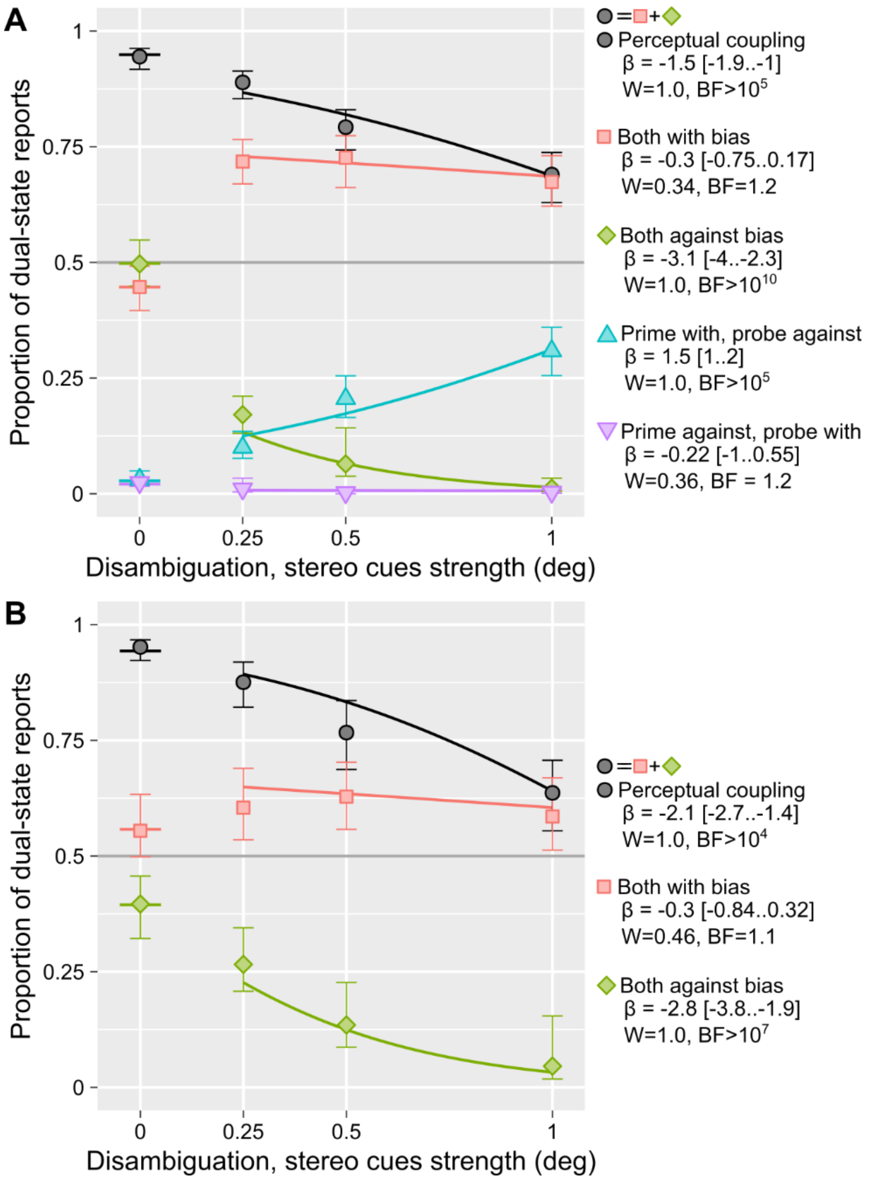

Figure S1. Experiment 1, perceptual coupling and dual-states for (A) parallel and (B) coaxial layout. The proportion of time the individual dual-states were dominant throughout the 60-second presentation as a function of disambiguation cues strength. Circles and error bars depict group mean and bootstrapped 95% bias-corrected accelerated confidence intervals. Solid lines show the prediction of a median model. The legend on the right shows the main effect of stereo strength on each dual state (for details see **Table S1**). β: median coefficient and 95% credible interval, W: relative weight of the full model compared to a reduced model without the stereo strength term (two weights add up to 1, weight above 0.5 indicates term significance), BF: Bayes Factor for the full versus reduced model.

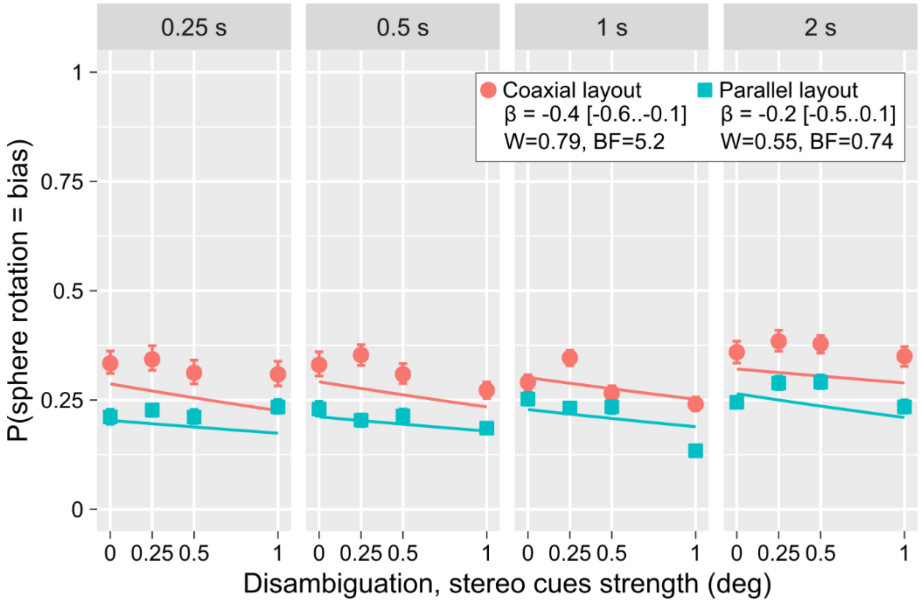

Figure S2. Experiment 2, Proportion of trials when both spheres corotated in the direction of bias (against the default rotation direction of the probe). See **Figure S1** for legend details and **Table S2** for full statistical analysis.

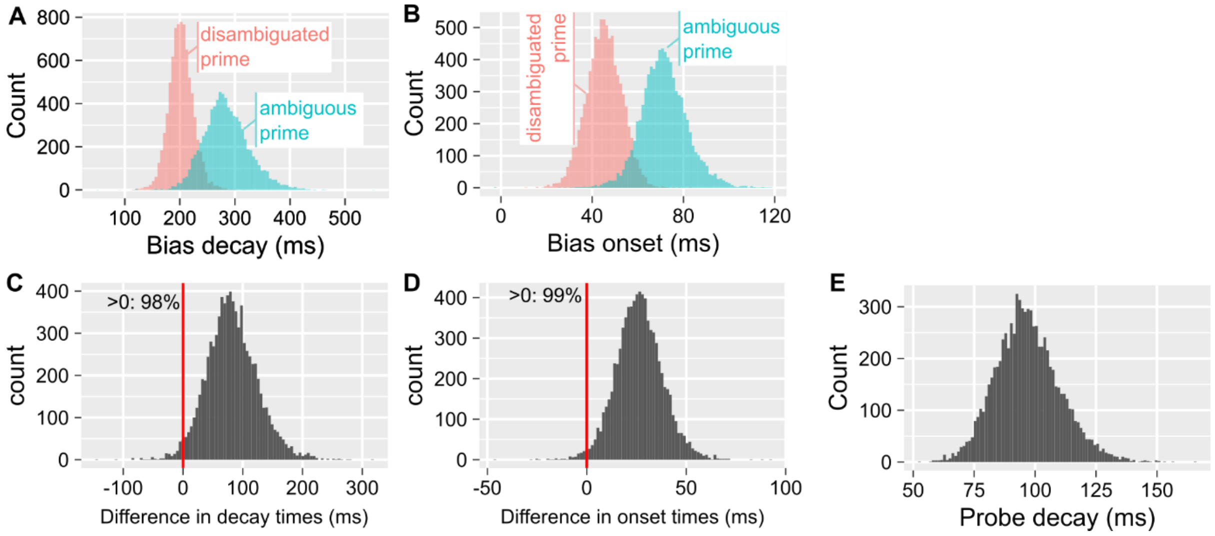

Figure S3. Experiment 3, posterior distributions for population-level model parameters. A) Bias decay time ($\mu_C^{\tau_{bias}}$). B) Bias onset time ($\mu_C^{T_{bias}}$). C) Difference of bias decay times for the prime conditions ($\mu_{ambiguous}^{\tau_{bias}} - \mu_{biased}^{\tau_{bias}}$). D) Difference of bias onset times for the prime conditions ($\mu_{ambiguous}^{T_{bias}} - \mu_{biased}^{T_{bias}}$). E) Probe decay time ($\mu^{\tau_{probe}}$).

Table S1. Experiment 1. A, B) Statistical analysis of perceptual dominance of dual states via hierarchical Bayesian GLM (beta family with logit link) with the presence of stereo cues (Stereo) and their strength (Strength) as fixed factors, and participants' identity as a random factor (intercepts only). C) Statistical analysis of the proportion of time the individual spheres rotated in the direction of bias via hierarchical Bayesian GLM (beta family with logit link) with the presence of stereo cues (Stereo) and their strength (Strength) as fixed factors, and participants' identity as a random factor (intercepts only). The table shows an estimate and 95% credible intervals for each term. *ΔWAIC* shows the difference in the expected log predictive density between the full model and a reduced model without the corresponding term (negative values indicate a preference for the full model with the term). *Weight* shows a relative weight of the full model. The two weights sum up to 1.0, values above 0.5 indicate that the factor substantially improves models' predictions. BF is the Bayes Factor for full versus reduced model, higher values indicate that the model with the term is preferred.

Dual state	Term	Estimate	95% CI	ΔWAIC	Weight	BF
A) Parallel layout						
**Overall coupling**	Stereo	-0.68	-1.17..-0.17	-2.98±2.82	0.95	26
	Strength	-1.45	-1.92..-0.98	-14.68±5.68	1.00	$>10^5$
**Both With**	Stereo	1.28	0.91..1.68	-17.21±5.09	1.00	$>10^6$
	Strength	-0.29	-0.75..0.17	0.65±1.42	0.34	1.2
**Both Against**	Stereo	-1.07	-1.66..-0.53	-7.53±3.19	1.00	$>10^3$
	Strength	-3.14	-4.03..-2.28	-25.15±6.94	1.00	$>10^{11}$
**Prime With, Probe Against**	Stereo	1.21	0.68..1.75	-9.39±4.11	1.00	$>10^3$
	Strength	1.54	1.07..2.02	-15.34±5.54	1.00	$>10^5$
**Prime Against, Probe With**	Stereo	-0.98	-1.62..-0.33	-3.41±2.89	0.97	60
	Strength	-0.22	-0.99..0.55	0.57±0.3	0.36	1.2
B) Coaxial layout						
**Overall coupling**	Stereo	-0.18	-0.85..0.51	1.09±0.73	0.25	0.97
	Strength	-2.05	-2.73..-1.35	-14.74±4.04	1.00	$>10^4$
**Both With**	Stereo	0.46	-0.04..0.96	-1.39±2.06	0.80	2.9
	Strength	-0.27	-0.84..0.32	0.18±1.14	0.46	1.1
**Both Against**	Stereo	-0.09	-0.78..0.58	0.76±0.38	0.32	0.81
	Strength	-2.86	-3.78..-1.94	-19.73±6.08	1.00	$>10^7$
C) Parallel layout, individual spheres						
**Prime sphere**	Stereo	1.17	0.61..1.74	-8.01±3.38	1	2,814
	Strength	2.97	2.11..3.85	-21.96±6.84	1	$>10^9$
**Probe sphere**	Stereo	1.22	0.85..1.61	-16.53±4.82	1	$>10^6$
	Strength	-0.32	-0.77..0.13	0.14±1.57	0.47	1.5

Table S2. Experiment 2. Effect of the strength of biasing cues (*Stereo Strength*), probe duration, probe SOA, and the interaction of cues strength and probe duration on the proportion of trials when A) each sphere was reported as rotating in the direction of the bias, parallel layout only, B) both spheres corotated in the direction of the bias. Bayesian GLMM (binomial model, participants' identity as a random factor, intercepts only). See **Table S1** for details.

Sphere or Layout	Term	Estimate	95% CI	ΔWAIC	Weight	BF
A) Parallel layout, the proportion of time sphere rotating in the direction of the bias						
**Prime**	Stereo Strength	1.079	0.607..1.565	-9.11±5.03	0.86	17,779
	Probe Duration	-0.472	-0.643..-0.306	-13.86±5.71	0.93	389,760
	Probe SOA	0.016	-1.475..1.498	0.88±0.1	0.00	1.93
	Strength:Duration	1.330	0.872..1.807	-15.27±6.7	0.84	>1e6
**Probe**	Stereo Strength	-0.225	-0.514..0.056	-0.44±1.57	0.68	1.16
	Probe Duration	0.155	0.016..0.288	-0.98±2.26	0.69	2.02
	Probe SOA	-0.573	-1.598..0.456	0.32±1.1	0.24	2.28
	Strength:Duration	-0.058	-0.301..0.185	1.09±0.49	0.00	0.34
B) Both layouts, the proportion of time both spheres corotated in the direction of the bias						
**Parallel layout**	Stereo Strength	-0.176	-0.468..0.115	-0.07±1.21	0.55	0.74
	Probe Duration	0.195	0.06..0.331	-3.25±2.78	0.92	8.14
	Probe SOA	-0.471	-1.456..0.581	0.54±0.89	0.00	1.89
	Strength:Duration	-0.063	-0.31..0.179	0.58±0.52	0.00	0.35
**Coaxial layout**	Stereo Strength	-0.346	-0.63.. -0.069	-1.55±2.31	0.79	5.18
	Probe Duration	0.092	-0.044.. 0.23	-0.06±1.3	0.54	0.40
	Probe SOA	-0.497	-1.562.. 0.593	0.38±0.92	0.05	2.13
	Strength:Duration	0.097	-0.14.. 0.344	0.68±0.78	0.00	0.45

Table S3. Experiment 3, the comparison of four models with different combinations of independent/common bias onset and decay times. The best model has both independent decay and onset times for ambiguous and disambiguated primes. ΔWAIC difference in expected log predictive density compared to the top model. Weight – model weight computed via stacking algorithm. BF – Bayes Factor comparing the top model to other models.

**Model**	**ΔWAIC**	**Weight**	**BF**
independent decay, independent delay		0.296	
common decay, independent delay	-3.6±2.9	0.287	$>10^6$
independent decay, common delay	-4.2±4.4	0.418	$>10^7$
common decay, common delay	-4.7±4.4	0	$>10^7$

Movie S1. Experimental stimuli.
